# Radical Cystectomy in England from 2013 to 2019 on 12,644 patients: An analysis of national trends and comparison of surgical approaches using Hospital Episode Statistics data

**DOI:** 10.1002/bco2.79

**Published:** 2021-03-12

**Authors:** Ashwin Sunil Tamhankar, David Thurtle, Alexander Hampson, Omar El‐Taji, Ramesh Thurairaja, John D. Kelly, James W. F. Catto, Tim Lane, James Adshead, Nikhil Vasdev

**Affiliations:** ^1^ Hertfordshire and Bedfordshire Urological Cancer Centre Lister Hospital Stevenage UK; ^2^ Department of Urology Guys Hospital London UK; ^3^ Division of Surgery and Interventional Science University College London London UK; ^4^ Academic Urology Unit University of Sheffield Sheffield UK; ^5^ School of Life and Medical Sciences University of Hertfordshire Hatfield UK

**Keywords:** bladder cancer, complications, cost, outcomes, radical cystectomy

## Abstract

**Introduction:**

We evaluate the data of 12,644 Radical Cystectomies in England (Open, Robotic and Laparoscopic) with trends in the adaption of techniques and post‐operative complications.

**Methods:**

This analysis utilised national Hospital Episode Statistics (HES) from NHS England.

**Results:**

There was a statistically significant increase (*P* < .001) in the number of Robotic assisted radical cystectomies from 10.8% in 2013‐2014 and 39.5% in 2018‐2019.The average LOS reduced from 12.3 to 10.8 days for RARC from 2013 to 2019 similarly the LOS reduced from 16.2 to 14.3 for ORC. The rate of sepsis (0‐90 days) did rise from 5% to 14.5% between 2013‐2014 and 2017‐2018 for the entire cohort (*P* < .001). Acute renal failure (ARF) increased over the years from 9.5% to 17% (*P* < .001). The rate for fever, UTI, critical care activity and ARF were higher for ORC than RARC (*P* < .001).The comparison of all episodes within 90 days for conduit versus non‐conduit diversions showed significantly higher rates of sepsis, infections, UTI and fever in non‐conduit group .Overall complications were significantly higher in non‐conduit group throughout the duration except was year 2016‐17(*P* < .001).The robotic approach has increased in last 5 years with nearly 40% of the cystectomies now being robotically in 2018‐19 from the initial percentage of 10.8% in 2013‐14.

**Conclusion:**

This evaluation of the HES data from NHS England for 12,644 RC confirms an increase in the adoption of Robotic Cystectomy. Our data confirms the need to develop strategies with enhanced recovery protocols and post‐operative close monitoring following Radical Cystectomy in order to reduce post‐operative complications.

## 1 INTRODUCTION

Open radical cystectomy (ORC) has evolved since its first description nearly 80 years ago.^1^ Perioperative outcomes have slowly improved, but overall, 90‐day complication rates have been reported to be as high as 65%.^2^ Morbidity can particularly correlate with urinary diversion technique.^3^ Robotic‐assisted laparoscopic techniques were first used for RC in 2003.^4^ and the feasibility of Robot‐Assisted Radical Cystectomy (RARC), has subsequently been demonstrated through randomized trials with potential advantages including reduced intra‐operative blood loss and shortened hospital stay.^5^, ^6^, ^7^, ^8^ The RAZOR trial indicated oncological equivalence of robotic and open cystectomy with respect to two‐year progression‐free survival.^9^, ^10^ The recently completed phase 3 iROC Trial which compared RARC with ORC will report length of stay as one of the primary outcome measures, providing further randomized data to inform comparisons.^11^

The robotic approach has received criticism due to cost implications and a lack of long‐term oncological data; however, there is now a consistent increase in uptake of minimally invasive surgical approaches, especially in RARC over the last decade.^12^, ^13^, ^14^ The European Association of Urology (EAU) in 2020 confirmed that RARC is a feasible and safe approach with comparable perioperative and long‐term complications to ORC (level of evidence 1b).^15^, ^16^, ^17^, ^18^ The uptake of RARC in clinical practice worldwide has been thought to be slower than elsewhere in the developed world due to concerns about cost‐efficiency.^19^, ^20^ However, uptake of RARC in the United Kingdom is relatively unexplored in the scientific literature, nor has its impact upon shorter term outcomes been hitherto assessed.^10^

We sought to evaluate Hospital Episode Statistics (HES) data from 2013 to 2019 for all radical cystectomies (RC) performed in England, with a specific focus on trends in the surgical approach and implications on early outcomes, re‐admissions, and interactions with the health care system within 90 days of surgery.

## 2 METHODS

This analysis utilized national Hospital Episode Statistics (HES) data from NHS England, containing information on inpatient admissions and outpatient appointments for all English NHS Clinical Commissioning Groups (CCGs)^21^ as reported at the time of patients’ interaction with the healthcare system. HES data were accessed using Harvey Walsh Health Informatics (Cheshire, UK) as a licensed intermediary. The work was supported by a research grant from Intuitive Surgical (California, USA). The data were pseudonymized at a source, precluding the need for ethical approval. Data are recorded on a real‐time basis, avoiding any potential recall bias. Each “episode” is defined as an inpatient admission during which patients are assigned a diagnosis coded for in the International Statistical Classification of Diseases and Related Health Problems, 10th revision (ICD‐10).^22^ The HES‐recorded procedure‐specific codes (Classification of Intervention and Procedure Codes or OPCS‐4) were used to identify patients from 2013 to 2019 and to classify each patient in to an operative group.^23^ The exact OPCS‐4 codes used are outlined in Annexure 1. Using this information patients were separated into three groups, namely ORC, Laparoscopic cystectomy, and RARC. Patients were also stratified according to urinary diversion technique, namely conduit or non‐conduit diversion.

Patients who were either readmitted or attended an accident and emergency department in the first 90 days following their index procedure were also identified, along with the ICD‐10 diagnosis code corresponding to the reason for readmission. These data were available from years 2013‐2019 only.

For the cost analysis, data for the cost per procedure as well as the cost of re‐admission per procedure were evaluated along with the global burden of expenditure in each modality for these past years.

### 2.1 Statistical analysis

Data were descriptively analyzed. Continuous variables were described using means and standard deviations and categorical variables were described using frequencies and percentages. A chi‐square test for trend in proportions was used to test the null hypothesis that there was no trend in the proportions over years against the alternative hypothesis that there was a linear trend in the proportion of patients undergoing cystectomy. The Mann‐Kendall test for trend was used to test the null hypothesis that there was no monotonic trend in the average length of stay (LOS) against the alternate hypothesis that a trend does exist. Intergroup comparison for 90 days post‐operative events in a particular year, along with entire duration, was performed with specific comparison between ORC and RARC cohorts. Intergroup analysis between different groups was performed by chi‐square test for trend in proportions. For cost analysis, the multivariate Mann‐Kendall test, an extension of Mann‐Kendall test for trend, was used to evaluate the global trend between the groups. For cost comparison pertaining to each specific complication, one‐way ANOVA test was used. Data were analyzed using RStudio Version 1.2.5019.

## 3 RESULTS

### 3.1 Demographics

From 2013 to 2019, HES data captured a total of 12,644 RC in England. Approximately 2,100 cystectomies were performed in each year. Overall, 9,056 patients were male (71.6%) and 3,588 (28.4%) were female. Only 1,118 (8.8%) patients were aged 50 years or under; 692 patients (5.5%) were aged over 80 years at the time of surgery. The proportion of male: female patients was consistent across the time period (*P* = .185).. The age of patients increased over time, with the proportion of patients aged 70 and above increasing from 35.5% in 2013‐14 to 38.4% in 2018‐19 (*P* < .001). There was also a significant decrease in the number of patients < 60 years undergoing cystectomy (*P* < .001). Within the entire cohort 38.6% of patients had neo‐adjuvant chemotherapy [36.6%—Laparoscopic Radical Cystectomy, 37.9%—Open Radical Cystectomy and 40.9%—Robotic Radical Cystectomy (*P* = .005)]. Demographic data and Baseline Characteristics (Table 1).

**TABLE 1:** Demographic data and baseline characteristics

Variable	Level	Overall	LAP	OPEN	RAS	*P*‐value
n		12,644	1,029	8,252	3,363	
Approach (%)	LAP	1,029 (8.1)				
	OPEN	8,252 (65.3)				
	RAS	3,363 (26.6)				
Gender (%)	Male	9,056 (71.6)	684 (66.5)	5,754 (69.7)	2,618 (77.8)	<.001
	Female	3,588 (28.4)	345 (33.5)	2,498 (30.3)	745 (22.2)	
IMD04 quartile (%)	High	3,389 (27.3)	232 (22.7)	2,173 (26.9)	984 (29.8)	<.001
	Median	3,503 (28.2)	320 (31.3)	2,264 (28.0)	919 (27.8)	
	Low	3,007 (24.2)	263 (25.8)	1946 (24.1)	798 (24.2)	
	Very Low	2,504 (20.2)	206 (20.2)	1697 (21.0)	601 (18.2)	
Year of operation (%)	2013/14	2,115 (16.7)	214 (20.8)	1673 (20.3)	228 ( 6.8)	<.001
	2014/15	2,102 (16.6)	210 (20.4)	1559 (18.9)	333 ( 9.9)	
	2015/16	2,100 (16.6)	165 (16.0)	1,448 (17.5)	487 (14.5)	
	2016/17	2068 (16.4)	142 (13.8)	1,236 (15.0)	690 (20.5)	
	2017/18	2093 (16.6)	172 (16.7)	1,167 (14.1)	754 (22.4)	
	2018/19	2,166 (17.1)	126 (12.2)	1,169 (14.2)	871 (25.9)	
Age band (%)	0‐30	190 (1.5)	66 (6.4)	115 (1.4)	9 ( 0.3)	<.001
	31‐40	260 (2.1)	52 (5.1)	180 (2.2)	28 ( 0.8)	
	41‐50	668 (5.3)	58 (5.6)	471 (5.7)	139 ( 4.1)	
	51‐60	1816 (14.4)	125 (12.1)	1,208 (14.6)	483 (14.4)	
	61‐70	4,349 (34.4)	308 (29.9)	2,801 (33.9)	1,240 (36.9)	
	71‐80	4,669 (36.9)	364 (35.4)	3,003 (36.4)	1,302 (38.7)	
	81+	692 ( 5.5)	56 ( 5.4)	474 ( 5.7)	162 ( 4.8)	
Ethnic group (%)	Asian	177 ( 1.4)	24 ( 2.3)	116 ( 1.4)	37 ( 1.1)	<.001
	Black	84 ( 0.7)	2 ( 0.2)	50 (0.6)	32 (1.0)	
	Other/Unknown	2,205 (17.4)	122 (11.9)	1,316 (15.9)	767 (22.8)	
	White	10,178 (80.5)	881 (85.6)	6,770 (82.0)	2,527 (75.1)	
CCI index (%)	1	6,092 (48.2)	512 (49.8)	3,976 (48.2)	1604 (47.7)	.001
	2	2,213 (17.5)	142 (13.8)	1516 (18.4)	555 (16.5)	
	3	4,339 (34.3)	375 (36.4)	2,760 (33.4)	1,204 (35.8)	
Prior chemotherapy (%)		4,882 (38.6)	377 (36.6)	3,130 (37.9)	1,375 (40.9)	.005

### 3.2 Surgical approach

**FIGURE 1:**
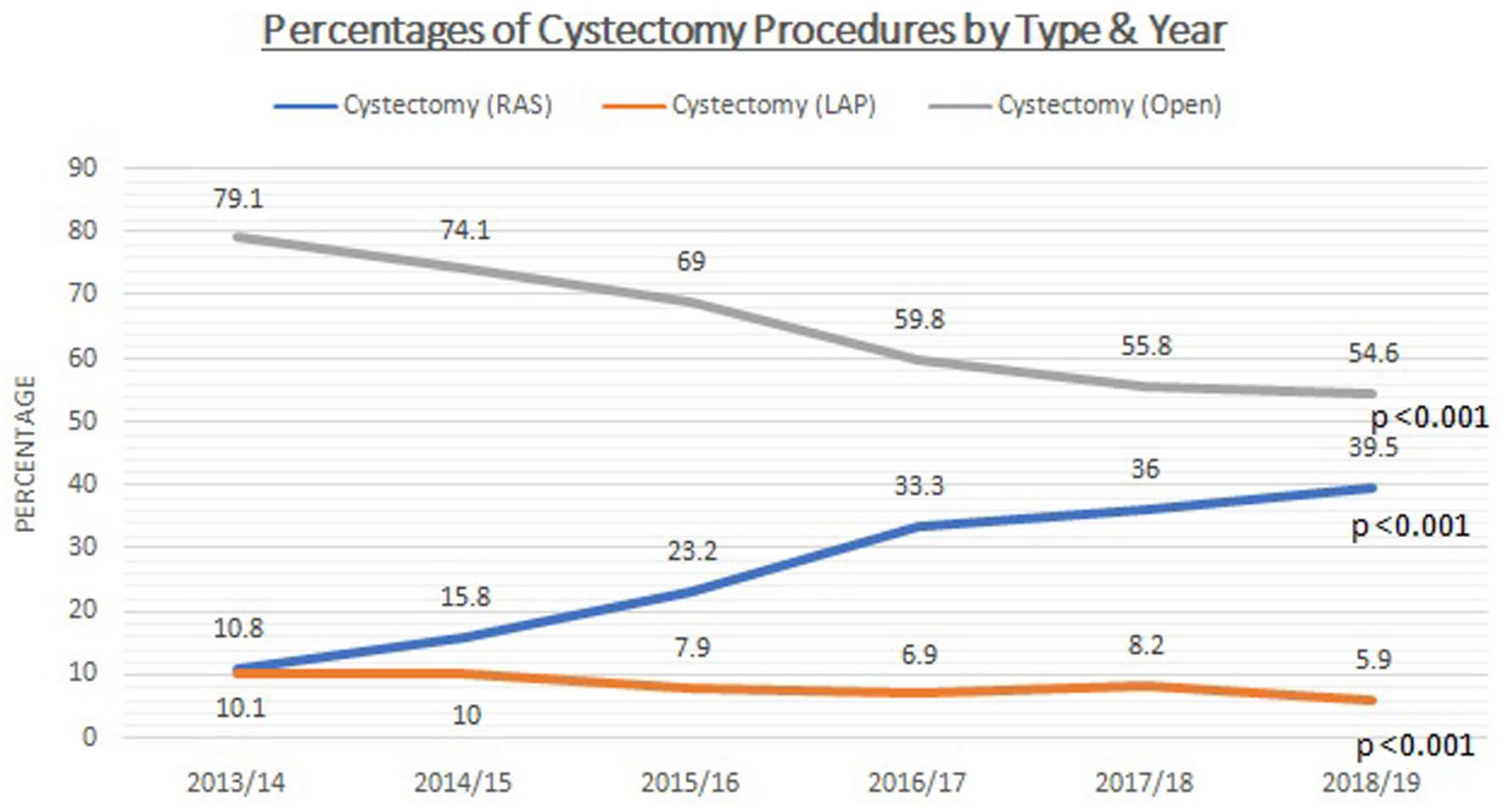
Trend in radical cystectomy (RC) surgical approach over time

Across the cohort, 8,252 (65.3%) cystectomies were performed as ORC, 3,363 (26.6%) as RARC, and 1,029 (8.1%) as laparoscopic assisted (LRC). There was a significant increase in the number of RC being performed robotically from 10.8% in 2013‐2014 to 40.2% in 2018‐19 (*P* < .001) (Figure 1). A reduction in the rate of both LRC (*P* < .001) and ORC (*P* < .001) was noted during the same time period. The proportion of ORC procedures fell from 79.1% in 2013‐14 to 54% in 2018‐19. The difference between the three approaches was significant both as a trend with respect to time and by intergroup comparison within each year (Table 2).

**TABLE 2:** Trends in radical cystectomy (RC) surgical approach over time

Cystectomy procedure type	Percentage of patients by cystectomy type						Comparison with respect to time
	2013/14	2014/15	2015/16	2016/17	2017/18	2018/19	*P*‐value
RAS	228 (10.8%)	333 (15.8%)	487 (23.2%)	690 (33.4%)	754 (36%)	871 (40.2%)	<.001
LAP	214 (10.1%)	210 (10%)	165 (7.9%)	142 (6.9%)	172 (8.2%)	126 (5.8%)	<.001
Open	1673 (79.1%)	1559 (74.2%)	1,448 (69%)	1,236 (59.8%)	1,167 (55.8%)	1,169 (54%)	<.001
Total procedures	2,115	2,102	2,100	2,068	2,093	2,166	
Intergroup comparison	<0.001	<0.001	<0.001	<0.001	<0.001	<0.001	

### 3.3 Length of stay

**FIGURE 2:**
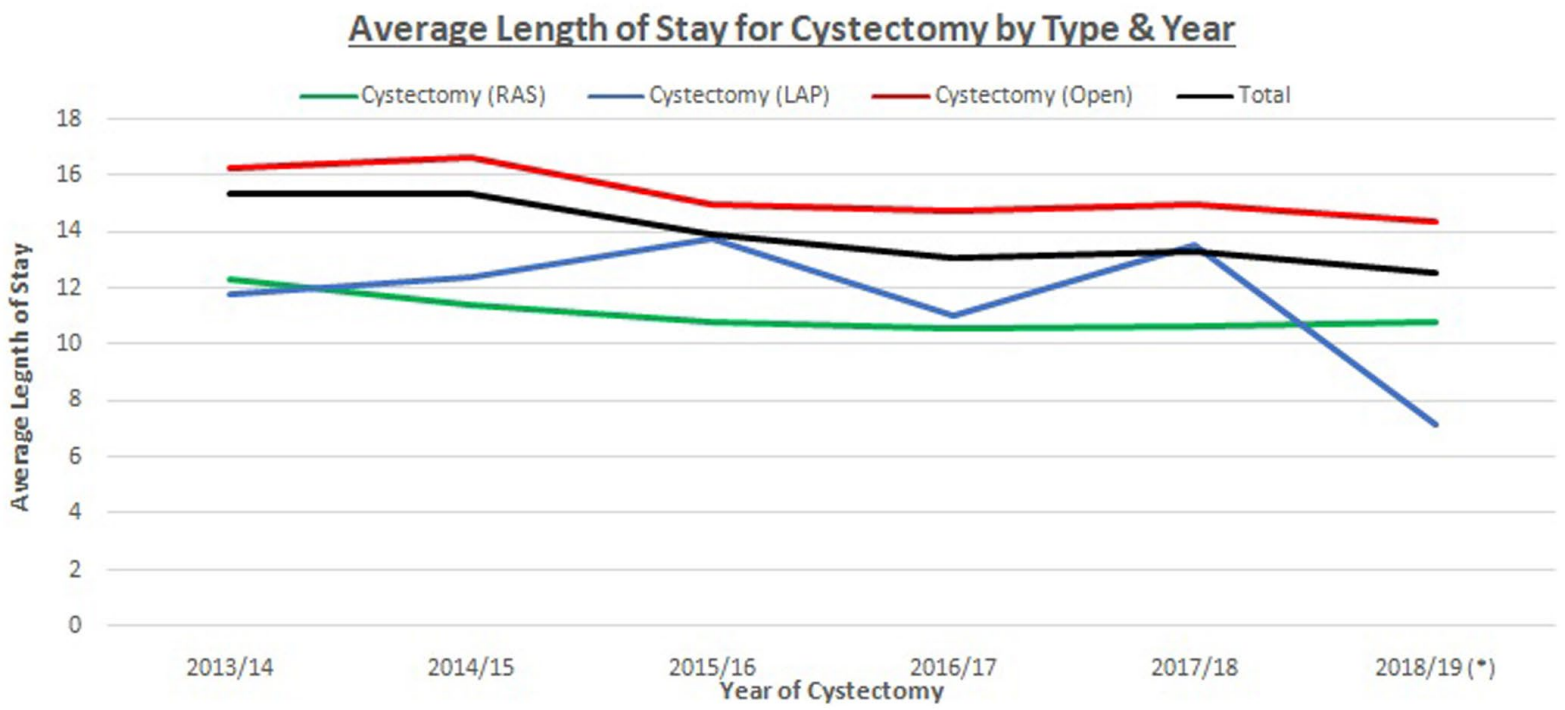
Average length of stay following each surgical approach to cystectomy over time

Mean LOS for the entire cohort was 12.5 days. Across all surgical approaches, there was a trend toward a decreased average length of spital stay (LOS) between 2013 and 2019 although this failed to reach statistical significance (Figure 2). The average LOS reduced from 12.3 to 10.8 days for RARC (*P* = .18) and from 16.2 to 14.3 days for ORC between the first and last time period, respectively.

Following propensity‐score matching, comparisons between RARC and ORC are shown in Table 2. Median LOS following RARC was 8 days for RARC, compared to 11 days for ORC (*P* < .001).

### 3.4 Post‐procedure events

Among all 12,644 patients, the proportion for whom there were recorded ICD codes for “post‐operative events” within 90 days were consistently in the region of 90%. as well as for The rate of recorded sepsis rose from 5% to 14.5% (*P* < .001) (Table 2). The recorded acute renal failure/acute kidney injury (AKI) increased over time from 9.5% to 17% (*P* < .001). The proportion of patients listed for critical care activity and cardiac episodes also rose (*P* < .001). However, 90‐day in‐hospital mortality was stable across time periods (*P* = .270). A trend toward increasing rates of recorded sepsis, fever, and acute renal failure was seen across all surgical approaches (Table 3). Comparisons of 90‐day events between RARC and ORC, following the propensity score matching, are outlined in Table 3.

**TABLE 3:** Length of stay (LOS), readmission, LOS following readmission, and post‐op events data comparisons for robotic‐assisted cystectomy (RARC) compared to open cystectomy (ORC) following propensity score matching, across the whole data period

		Overall	LRC	ORC	RRC	*P* value
Index hospitalization LOS (median, IQR)		10.00 [7.00, 15.00]	9.00 [6.00, 13.00]	11.00 [8.00, 17.00]	8.00 [6.00, 12.00]	<.001
90 day (non‐elective) readmission (any diagnosis)		3,274 (25.9)	218 (21.2)	2078 (25.2)	978 (29.1)	<.001
Average length of stay for readmission for cystectomy with a 90 day (NEL) readmission (any diagnosis) (median, IQR)		3.00 [1.00, 7.00]	2.00 [1.00, 6.75]	4.00 [1.00, 8.00]	3.00 [1.00, 7.00]	.001
Other events (%)	Patients (with any of the events listed)	11,490 (90.9)	809 (78.6)	7,627 (92.4)	3,054 (90.8)	<.001
	Sepsis	1,083 ( 8.6)	67 ( 6.5)	721 ( 8.7)	295 ( 8.8)	.049
	Other infections	1584 (12.5)	69 ( 6.7)	1,156 (14.0)	359 (10.7)	<.001
	Cardiac events	7,583 (60.0)	561 (54.5)	5,008 (60.7)	2014 (59.9)	.001
	Critical care activity	9,474 (74.9)	583 (56.7)	6,457 (78.2)	2,434 (72.4)	<.001
	Ventilation	570 ( 4.5)	36 ( 3.5)	422 ( 5.1)	112 ( 3.3)	<.001
	Acute renal failure	1797 (14.2)	137 (13.3)	1,149 (13.9)	511 (15.2)	.142
	Post Op complications	2,938 (23.2)	191 (18.6)	1962 (23.8)	785 (23.3)	.001
	Fever	429 ( 3.4)	26 ( 2.5)	300 ( 3.6)	103 ( 3.1)	.084
	UTI	1896 (15.0)	118 (11.5)	1,314 (15.9)	464 (13.8)	<.001
	Death (In Hospital)	321 ( 2.5)	20 ( 1.9)	225 ( 2.7)	76 ( 2.3)	.157
Index hospitalization cost (median, IQR, mean, SD)		9,256.60 [7,385.25, 10,669.42]	8,390.09 [7,164.35, 10,047.37]	9,229.87 [7,338.39, 10,386.96]	9,571.53 [7,714.51, 11,186.52]	<.001
		8,885.41 (3,883.99)	7,912.25 (4,120.34)	8,697.86 (4,163.33)	9,651.76 (2,822.10)	<.001
30 day cost (median, IQR, mean, SD)	*Inclusive of index hospitalization cost*	9,711.24 [7,513.34, 11,248.24]	8,812.75 [7,186.84, 10,456.45]	9,686.90 [7,436.28, 11,025.25]	10,191.70 [8,205.56, 11,961.83]	<.001
		9,580.22 (4,879.01)	8,433.52 (4,991.19)	9,390.74 (5,264.42)	10,404.55 (3,550.68)	<.001
90 day cost (median, IQR, mean, SD)	*Inclusive of index hospitalization cost*	10,015.66 [7,672.51, 12 179.01]	9,144.79 [7,281.97, 10,984.59]	9,967.36 [7,546.56, 12,037.42]	10,669.42 [8,361.28, 12,777.59]	<.001
		10,512.41 (5,813.69)	9,207.70 (5,885.50)	10,365.12 (6,186.41)	11,280.34 (4,616.07)	<.001
Inpatient cost 90 day (median, IQR, mean, SD)	*Inclusive of index hospitalization cost*	10,040.64 [7,703.42, 12,190.25]	9,144.79 [7,281.97, 10,985.28]	9,995.17 [7,604.93, 12,077.96]	10,669.42 [8,361.28, 12,777.59]	<.001
		10,585.68 (5,768.67)	9,216.65 (5,881.35)	10,474.64 (6,128.47)	11,280.34 (4,616.07)	<.001
Outpatient cost 90 day (median, IQR, mean, SD)	*Inclusive of index hospitalization cost*	104.59 [0.00, 244.90]	0.00 [0.00, 0.00]	104.59 [0.00, 256.71]	NA [NA, NA]	.293
		202.26 (327.02)	0.00 (NA)	204.56 (328.17)	NaN (NA)	NA

The proportion of patients undergoing ORC that required critical care activity increased over time (*P* < .001) and had an increased rate of cardiac events (*P* < .007) (Table 3). Between surgical approaches, there was a consistently higher rate of infection and colostomy among patients undergoing open procedures compared to robotic or laparoscopic (*P* < .001).

Following propensity score matching, the rates of critical care activity, were significantly lower for RARC patients, compared to ORC (*P* < .001), and were lower still for patients undergoing laparoscopic RC (*P* < .001) . Colostomy rates were 11.9% for ORC, compared to 7.8% for RARC (*P* < .001).

When comparisons were made between those with conduit vs non‐conduit diversions, significantly higher rates of sepsis, infections, UTI, and fever were seen in the non‐conduit group. Overall complications were significantly higher in the non‐conduit group throughout the time period (*P* < .001).

Table 3 Length of stay (LOS), readmission, LOS following readmission, and post‐op events data comparisons for robotic‐assisted cystectomy (RARC) compared to open cystectomy (ORC) following propensity score matching, across the whole data period.

### 3.5 Readmissions

The overall rate of 90‐day non‐elective readmissions (NER) across our entire cohort was 25.9%. Overall there was a slow increase in NER over time from 23.9% to 28.2% (*P* < .001). NER for the RARC group fell over time (*P* < .001) but started at a relatively high rate of readmission of 37.7% in 2013‐14. Conversely, there was an increasing trend of NER following ORC, but the NER rate was low at the start of the time period (22.4%) NER for LRC was fairly low throughout, ranging between 13.7% and 27.3% in any year (Table 4). Following propensity score matching, the rate of 90‐day NER was significantly lower for LRC than either RARC or ORC (*P* = .015).

**TABLE 4:** Cost analysis comparing LRC, ORC, and RRC

HRG description	LAP			Open			RAS		
	Patients	Mean cost	Median cost	Patients	Mean cost	Median cost	Patients	Mean cost	Median cost
Cystectomy with Urinary Diversion and Reconstruction with CC	389	£8,048	£9,272	3,492	£8,924	£9,947	1,074	£9,943	£10,108
Cystectomy with Urinary Diversion and Reconstruction without CC	220	£5,365	£7,346	1,265	£6,119	£7,420	395	£7,608	£7,774
Complex Open Bladder Procedures with CC Score 3+	79	£11,856	£11,097	580	£12,339	£11,103	814	£12,141	£11,440
Complex Open Bladder Procedures with CC Score 0‐2	42	£7,486	£7,361	286	£7,702	£7,381	641	£7,777	£7,629
Cystectomy with Urinary Diversion and Reconstruction, with CC Score 3+	69	£10,132	£9,763	787	£10,335	£9,895	66	£10,248	£9,796
Complex Open Bladder Procedures without Major CC	84	£6,467	£7,304	630	£6,223	£7,331	194	£7,180	£7,662
Cystectomy with Urinary Diversion and Reconstruction, with CC Score 0‐2	105	£7,588	£7,350	595	£7,166	£7,370	67	£7,637	£7,389
Complex Open Bladder Procedures with Major CC	29	£12,913	£11,719	300	£11,952	£11,834	70	£12,713	£12,272
Total Pelvic Exenteration				154	£15,326	£15,450			
Urinary Diversion without Cystectomy with Malignancy	9	£8,087	£8,675	133	£7,733	£8,797			
Complex Open, Upper or Lower Genital Tract Procedures				34	£8,507	£8,150			

### 3.6 Cost implications

The average cost per patient for RARC was £10,225 in the year 2018/19 whereas the average cost per open cystectomy spell was £ 9,975 for the year 2018/19. The variation over these six years did not show any significant difference in either of the groups (*P* = .707 and *P* = .450).] The intergroup comparison for the statistics for each year showed higher cost for RARC in comparison to ORC; however, global comparison for the trend did not show any significant variation (*P* .431). Average cost per patient per re‐admission did not vary with the time for any of the approaches RARC (*P* = .707), LRC (*P* = .259), and ORC (*P* = 1.00). Nearly £2,500 was required to be spent on managing the NEL for ORC or RARC. The mean cost for managing sepsis within 1‐90 days showed a non‐significant increasing trend from 2013 to 2018 for all three groups (Table 4).

## 4 DISCUSSION

This analysis of the HES data for six consecutive years from NHS England provides information on the trends in health care utilization for RC. The use of the robotic approach has increased over the past six years, with nearly 40% of cystectomies being performed robotically in 2018‐19 compared to 10.8% in 2013‐14. This large dataset of 12,644 RC procedures may help to increase confidence in switching toward robotic‐assisted or minimally invasive approaches in line with recent changes in international guidelines.^17^, ^24^

Bladder cancer is the second most common urological cancer in the United Kingdom, with an estimated annual incidence rate of 10,200 new cases every year.^25^ Mortality due to bladder cancer is high with 5,400 deaths annually in the United Kingdom, 3% of all cancer deaths. It is the seventh most common cause of cancer‐related death in males.^25^, ^26^ Significant debate surrounds the optimum surgical approach in the management of bladder cancer, including debates around the optimum outcome measures to use when assessing differences between approaches—which would include oncological, functional, and quality of life (QOL) related parameters.

The rate of uptake of RARC has increased globally; in the United States up to nearly one‐quarter of cystectomies were performed robotically as early as 2013.^13^ Uptake in the NHS was slower according to this dataset, perhaps due to a need for significant levels of published evidence on efficacy and cost viability to enable NHS funding. As highlighted in the clinical commissioning policy from NHS England, guidance did not support robot‐assisted procedures for bladder cancer until July 2016.^27^ Though robot‐assisted laparoscopic prostatectomy accounted for nearly 80% of UK prostatectomies in 2016, surgeons performing RC appear to have been slower to have embraced a robotic approach (10,27) This analysis shows RARC is becoming more widely utilized in England.^10^ We demonstrate that length of stay (LOS) shows a declining trend across all surgical approaches, including following RARC, for which an average LOS of 10.8days was recorded in 2018‐19 which is comparable to the established literature.^28^ LOS following RARC was shorter than ORC. Other factors can influence and impact the length of stay in NHS practice such as social factors which may affect the ability to compare this data internationally. LOS reductions across all surgical approaches may well be related to enhanced recovery protocols post‐surgery, upon which there has been significant emphasis.. The apparently higher rates of colostomy, infection, LOS, and readmission in ORC may be related to more complex cases or more advanced disease. Without data on tumor characteristics, we cannot fully adjust for this here. However, we noted that comorbidity rates were similar in each surgery type.

Non‐elective readmissions are a major factor in the cost effectiveness of cystectomy approaches. We observed that the NER rate following RARC declined, and the rate following ORC increased over our study period. However, given their respective starting points, they converged toward a similar rate of around 25%. This may be due to the fact that RARC was in its early days of adoption in the United Kingdom in the initial years of data collection, whereas ORC was well‐established. Alternatively, it may relate to changes in patient selection over time. Similar comparisons in other datasets have shown no major difference between the surgical approaches in terms of 30‐day readmission rates,^13^, ^29^ although RC it is the authors’ opinion that readmission rates should be quoted until at least 90‐days, as per our analysis. Of note, any in‐person interactions between a patient and the health care system in the post‐operative period will be reflected as NER in HES coding which may, for example, include attendances for catheter complications or stoma nurse clinic visits. It is reassuring that despite this potential over‐estimation, overall 90‐day NER rates in the United Kingdom are comparable to global data.

It is interesting to note the overall rate of recorded sepsis within 90‐days post cystectomy increased across our study period. This appears unlikely to be due to clinical reasons, but rather, may be an effect of increased awareness and reporting of the condition. The highest rate recorded was in 2015‐2016, corresponding with an NHS campaign in 2015 to detect and treat sepsis early.^30^ The American College of Surgeons National Surgical Quality Improvement Project database emphasizes that post RC, 25% of patients develop infections within 30 days with rates of sepsis being 12.7%.^31^ That study reported that operative time >480 minutes was associated with surgical site infections (SSI), sepsis, and UTI and that perioperative blood transfusion positively correlated with higher rates of SSI and sepsis.^31^ Indeed, it has been previously established that higher blood loss and the requirement of transfusions are more common in ORC than RARC, which was not further assessed in our work.^9^, ^17^, ^28^ Other work has shown that operative times may reduce over a surgeon's learning curve, or may be lower in high‐volume centers which may help minimize the infection‐risk post RC.^31^, ^32^ Antibiotic strategies and levels of resistance may also play a role in this apparent increase in the infections reported by HES. Similarly, increased rates of AKI throughout the cohort may be related to changing awareness, and to evolving electronic blood reporting systems which may translate to increased coding of such conditions.

Post‐operative complications within 90‐days remained relatively stable across the study period, and comparisons between ORC and RARC failed to demonstrate any difference (*P* = .599). Systematic reviews have reported that 90‐day grade 3 complication rates favor RARC whereas high‐grade complications are comparable between RARC and ORC.^28^ Other studies report in favor of RARC or equivalence when compared to ORC taking in terms of complications, but randomized trial data reported equivalent results for this metric.^9^, ^33^, ^34^

Non‐conduit diversions recorded significantly higher rates of sepsis, infections, UTI, fever, and complications compared to conduit diversions in our study, which is in keeping with published data (34). The data were limited in terms of comparing subgroups of diversion techniques within each surgical approach. Longer operating time requirements, the need for catheters and stents and more anastomoses may be some of the reasons for higher infections in the non‐conduit group. Another noteworthy detail from our study is the increasing number of older patients undergoing RC, with 38.4% of patients being > 70 years in 2018‐19. This may have implications on post‐operative recovery, as well as altered susceptibility to infections in the post‐operative phase.

Increasing uptake of RARC may have cost implications, which we have not explored thoroughly in this study. Published systematic review analyses have concluded RARC may be cost‐effective compared to ORC.^35^ Cost factors involved are predominantly based on equipment and operating room costs, length of stay, transfusion‐related requirements, complications, and readmission‐related expenditure.^35^ Effective ways to mitigate the potential higher cost of RARC may, therefore, be to reduce operative theater time, and increase the number of cases performed.^35^ Potential savings in RARC may be gained by the reduced burden on health care for readmissions.^36^ If the trend in reduced readmission rates following RARC continues, the cost‐effectiveness analyses may move in favor of RARC. Additionally, the healthcare utilizations for managing sepsis, infections, UTI, fever, critical care activity, and acute renal failure, which stand significantly higher in ORC than RARC, may result in some downstream saving.^37^, ^38^, ^39^

Particular strengths of this analysis relate to the large cohort size which represent real‐world data from patients undergoing cystectomy. HES data is objectively recorded by professional coders who are outside of surgical teams. This data is particularly useful for studying readmission data within a single nationalised health service, which allows for broad comparison of trends.

We do, however, recognize potential limitations inherent to observational data, and to HES data specifically. HES data may be subjected to incorrect coding and overlapping terms such as UTI, infection, and sepsis resulting in skewed data. Accuracy of recording over time may not be consistent, and cannot be controlled for. This study was unable to assess functional and oncological outcomes, as no data are collected on this via the HES system, which could have helped contextualize metrics we have assessed such as LOS and readmission rate. Also, we were unable to report, or adjust for many patient or tumor characteristics. Propensity score‐matching also has its limitations, particularly with regards to comparisons to the relatively small laparoscopic cohort. Nonetheless, the large sample size helps counteract some of these limitations and allows broad comparisons to be made.

## 5 CONCLUSION

This evaluation of NHS England HES data for 12,644 RC confirms a continued rise in the proportion of cystectomy being performed robotically. This paper emphasizes the need to further‐develop enhanced recovery protocols and close post‐operative monitoring of patients following radical cystectomy. RARC appears to have potential real‐world benefits of reduced LOS and reduced rates of many 90‐day post‐procedural events including infection, cardiac events, renal failure, and critical care activity.

## CONFLICT OF INTEREST

Nothing to disclose.

## ANNEXURE 1.

The HES recorded procedure‐specific codes‐Classification of Intervention and Procedure Codes (OPCS‐4) and International Statistical Classification of Diseases and Related Health Problems, 10th revision (ICD‐10).
